# Maternal Diet-Induced Obesity Alters Mitochondrial Activity and Redox Status in Mouse Oocytes and Zygotes

**DOI:** 10.1371/journal.pone.0010074

**Published:** 2010-04-09

**Authors:** Natalia Igosheva, Andrey Y. Abramov, Lucilla Poston, Judith J. Eckert, Tom P. Fleming, Michael R. Duchen, Josie McConnell

**Affiliations:** 1 Division of Reproduction and Endocrinology, King's College London, London, United Kingdom; 2 Department of Molecular Neuroscience, Institute of Neurology, University College London, London, United Kingdom; 3 School of Medicine, University of Southampton, Southampton, United Kingdom; 4 School of Biological Sciences, University Southampton, Southampton, United Kingdom; 5 Department of Cell and Developmental Biology, University College London, London, United Kingdom; Institute of Preventive Medicine, Denmark

## Abstract

The negative impact of obesity on reproductive success is well documented but the stages at which development of the conceptus is compromised and the mechanisms responsible for the developmental failure still remain unclear. Recent findings suggest that mitochondria may be a contributing factor. However to date no studies have directly addressed the consequences of maternal obesity on mitochondria in early embryogenesis.

Using an established murine model of maternal diet induced obesity and a live cell dynamic fluorescence imaging techniques coupled with molecular biology we have investigated the underlying mechanisms of obesity-induced reduced fertility. Our study is the first to show that maternal obesity prior to conception is associated with altered mitochondria in mouse oocytes and zygotes. Specifically, maternal diet-induced obesity in mice led to an increase in mitochondrial potential, mitochondrial DNA content and biogenesis. Generation of reactive oxygen species (ROS) was raised while glutathione was depleted and the redox state became more oxidised, suggestive of oxidative stress. These altered mitochondrial properties were associated with significant developmental impairment as shown by the increased number of obese mothers who failed to support blastocyst formation compared to lean dams. We propose that compromised oocyte and early embryo mitochondrial metabolism, resulting from excessive nutrient exposure prior to and during conception, may underlie poor reproductive outcomes frequently reported in obese women.

## Introduction

Obesity and related metabolic disorders are a major health issue worldwide. With increasing prevalence in all populations and age groups, the proportion of women of reproductive age who are obese is rising [1]. Evidence is growing that excessive body fat has a detrimental effect on female fertility and pregnancy [2]. Obese women take longer to conceive and have a higher risk of miscarriage compared to lean women [3].Obesity also impairs the immediate outcome of assisted reproductive technologies suggesting that maternal body mass index (BMI) may influence the potential for fertilisation and viability of early embryos [4]. Since the earliest stages of embryo development are primarily controlled by the oocyte, it is likely that a sub-optimal environment within the ovary and/or oviduct accounts for these poor reproductive outcomes.

As recently reviewed [5], understanding of the effects of maternal obesity on the structure and metabolism in oocytes and pre-implantation embryos is very limited. It has been proposed that a high plane of nutrition might lead to excessive enrichment of the reproductive milieu [6]. This in turn may induce alterations in oocyte metabolism and impede embryonic development. Indeed several studies have shown that abnormally high or low rates of metabolism may compromise oocyte and embryo development [7], [8]. Mitochondria are likely candidates for compromised metabolism in the embryo; these organelles are exclusively maternal in origin, and thus a deleterious influence of maternal BMI on mitochondria in the oocyte would strongly influence embryonic metabolism. Mitochondria also perform numerous regulatory functions during oocyte maturation [9], fertilization, initiation and progression of preimplantation embryos [10]. As energy producer, the central and most important function of mitochondria is the synthesis of adenosine triphosphate (ATP) by oxidative phosphorylation, a mechanism coupling the oxidation of nutrients and reducing equivalents (NAD(P)H, FADH_2_) with the phosphorylation of adenosine diphosphate. Both cytosolic and mitochondrial sources of NAD(P)H along with mitochondrial FADH_2_ stimulate the mitochondrial electron transport chain to pump H out of the mitochondrial matrix thereby hyperpolarising the inner mitochondrial membrane and generating the proton-motive force used to generate ATP. Electron donors NAD(P)H and FADH_2_ besides being used for energy production set the intracellular redox state. NADH oxidation in the mitochondria will produce ROS whereas NADPH oxidation (in the cytosol and mitochondria) serves to rejuvenate the antioxidant defence by reducing peroxiredoxins, thioredoxin and oxidised glutathione. Mitochondrial functions have, therefore, a dual impact on the intracellular redox state via regeneration antioxidant systems and via ROS production [11].

Mitochondria not only supply cells with their ATP, but are also the source of cellular guanosine-5′-triphosphate (GTP) as well as site of amino acid synthesis and reservoir of cell calcium. Thus, changes in mitochondrial activity can alter cell function in dramatic way. The importance of mitochondria in oocyte quality and embryo development is highlighted by reports showing that defects in mitochondrial biogenesis together with insufficient mitochondrial mass are associated with oocyte maturation failure and abnormal embryo development [12], [13]. Both the quality and quantity of mitochondria are therefore an essential prerequisite for successful fertilization and embryo development [14].

Studies *in vitro* have also highlighted the susceptibility of mitochondria within the oocyte and developing embryo to environmental stressors and have shown that even low-level acquired mitochondrial injuries may persist into embryonic life [15], [16]. Potential influences of maternal nutritional status in obesity are indicated by reports showing that periconceptual exposure to high energy substrates such as fatty acids [17] and proteins [18] results in perturbed oocyte and embryo mitochondrial metabolism. Hitherto, mitochondrial abnormalities of the oocyte and early embryo have not been identified as a direct consequence of maternal obesity. Using an established murine model of maternal diet induced obesity [19] and a live cell dynamic fluorescence imaging techniques coupled with molecular biology we have investigated the effects of maternal obesity on mitochondria l metabolism and biogenesis in oocytes and pre-implantation embryos. Our study is the first to show that maternal obesity during the periconceptional period resulted in an increased mitochondrial potential, biogenesis and damaging level of ROS in oocytes and zygotes. These changes were associated with reduced fertility and impaired embryo viability. Our findings have identified altered mitochondrial status as one of the probable mechanisms of obesity-associated reproductive and developmental failure.

## Results

### Maternal metabolic profile

At conception, female mice fed an obesogenic diet had a 43% increase in body weight, 2.5 fold increase in fat pad and a significantly higher concentration of serum fatty acids (p<0.05) compared with chow fed controls (Table 1). Although maternal serum leptin concentrations were similar in obese and lean mice, the concentration of leptin in the oviductal fluid was significantly elevated in obese females in comparison with lean females (p<0.05).

**Table 1:** Maternal weights and metabolic parameters.

Parameter	Control females	Obese females	Significance
Body weight (g)	22.9±1.2	32.8±1.3	P<0.001 (10)
Fat pads weight (g)	0.91±0.01	2.32±0.01	P<0.01 (10)
Serum glucose (mmol/L)	6.68±0.81	8.99±0.95	P>0.05 (10)
Serum FFA (mmol/L)	0.67±0.06	0.89±0.08	P<0.05 (10)
Serum triglycerides (mmol/L)	0.93±0.08	0.94±0.09	P>0.05 (10)
Serum leptin (pg/ml)	1172±270	1492±170	P>0.05 (8)
Oviductal leptin (pg/ml)	1067±293	1916±221	P<0.05 (7)
Oviductal glucose (mmol/l)	6.11±0.72	10.90±2.01	P>0.05 (7)

Data expressed as mean ± SEM. All serum measurements were fasting. Values in parentheses indicate n/group.

### Mitochondrial status in oocytes and zygotes of obese female mice

Live cell dynamic fluorescence imaging was employed to study effects of maternal obesity on mitochondrial function in oocytes and embryos. This technique together with a range of targeted fluorescent probes permitted comprehensive evaluation of mitochondrial function with simultaneous measurement of multiple mitochondrial variables in a single oocyte and embryo. The common vital mitochondrial membrane–specific dyes; MitoTrackers and JC1 have been extensively used to study mitochondrial dynamics and function in oocytes and embryos. However, prolonged excitation of cells loaded with MitoTrackers may impair mitochondrial function [20] whereas the JC1 dye appears sensitive to factors other than inner mitochondrial membrane potential (Δψm) [21] and may inhibit mitochondrial complex 1 [22]. We therefore chose to measure Δψm in single oocytes and embryos using a low toxicity potentiometric fluorescent dye-tetramethyl rhodamine methyl ester (TMRM; [23]).

**Figure 1:**
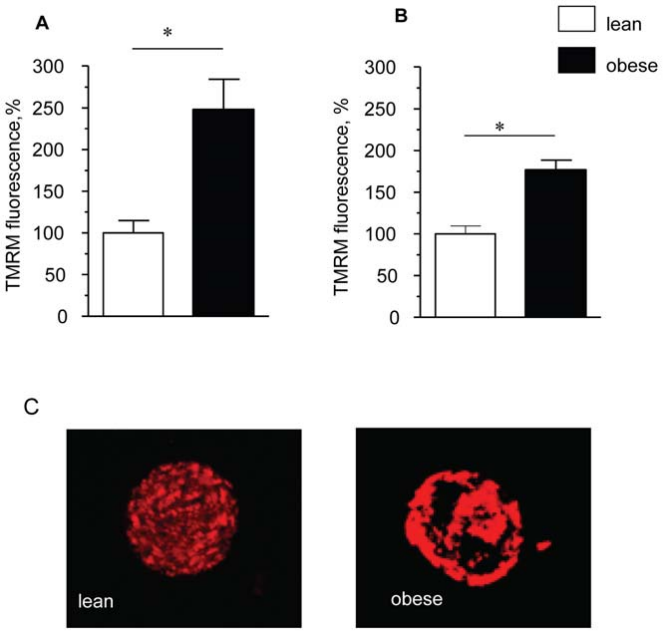
Maternal diet-induced obesity leads to increased mitochondrial activity in the oocytes and zygotes. The measurements of inner mitochondrial membrane potential (Δψ_m_) were made using confocal imaging of TMRM fluorescence. The signal intensity was quantified per pixel in a confocal slice after thresholding to remove background signal. (A) – oocytes and (B) zygotes derived from lean (n = 15 cells/group) and obese females (n = 15 cells/group). Relative intensity of TMRM fluorescence is expressed as a percentage of the signal from oocytes of lean mice. (C) Representative confocal images of mitochondria distribution in oocytes from lean and obese mice. * p<0.05. Data are mean ± SEM.

Maternal diet-induced obesity led to a dramatic increase in Δψm in oocytes and zygotes. The intensity of mitochondrial localised TMRM fluorescence in oocytes and zygotes of obese females increased by 147% (p<0.01) and 74% (p<0.01), respectively, compared with oocytes and zygotes of lean females (Figure 1A and 1B). Eggs from obese mice also had a different pattern of mitochondrial distribution as visualised by the distribution of TMRM staining (Figure 1C and 1D). Mitochondria were distributed evenly throughout the ooplasm of the eggs from lean females. In contrast, mitochondria in eggs from obese females had a discontinuous distribution with high density clusters localised to the cortical ooplasm and surrounding the nucleus. Since the organisation of mitochondria within cells is important for the signalling events associated with fertilisation [10], this abnormal localisation of mitochondria may be detrimental for the preimplantation embryo. Indeed, a similar aggregated mitochondrial structure was identified in preimplantation embryos undergoing arrest [24].

**Figure 2:**
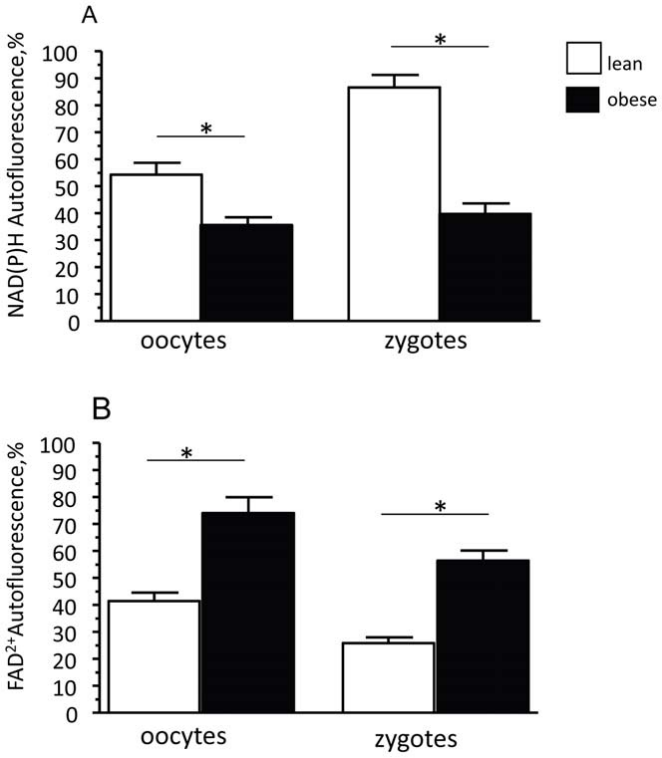
Maternal diet-induced obesity is associated with an oxidised intracellular redox state in oocytes and zygotes. The redox state of single oocytes and zygotes from lean (n = 15 cells/group) and obese (n = 15 cells/group) mice was estimated through measurements of NAD(P)H and FAD^2+^ autofluorescence intensity. The resting redox state is defined as a ratio of the maximally oxidised (response to 1 mM FCCP) and maximally reduced (response to 1 mM NaCN) signals. The fluorescence signals are normalised between 100 and 0. For NAD(P)H autofluorescence (A): 0 - maximally oxidised state; 100 – maximally reduced state. This scale is reversed for FAD^2+^ fluorescence (B). 0 – maximally reduced state; 100 – maximally oxidised state. * p<0.05 relative lean group. Data are mean ± SEM.

An increase in Δψm may have a number of origins including a higher supply of substrates and increased mitochondrial respiratory activity or inhibition of ATP synthase activity [25]. To differentiate between different mechanisms we analysed the redox state in oocytes and zygotes, measured as the autofluorescence signal derived from NAD(P)H or FAD^2+^ [23]. Confocal laser-scanning microscopy (CLSM) revealed that the NAD(P)H in oocytes and zygotes from obese mice was more oxidised than in eggs from lean mice (oocytes - 36%±3 vs 55%±4, p<0.05; zygotes – 27%±2 vs 86%±5, p<0.05 on a scale that runs from 0% for full oxidation to 100% for full reduction) (Figure 2A). Imaging FAD^2+^ autofluorescence also revealed that flavoproteins were more oxidised in oocytes and zygotes from obese females compared to eggs from lean females (oocytes – 74%±6 vs 41%±3, p<0.05, zygotes- 56%±4 vs 40%±4, p<0.05: note that for this signal, 100% is fully oxidised and 0%, fully reduced) (Figure 2B). These changes indicate increased oxidation of the pyridine nucleotide and flavoprotein pools [23] and suggest a shift in the intracellular redox status towards oxidation in the eggs from obese mice.

Studies in mitochondria respiratory chain function *in vivo* [26] and *in vitro* [11], [23] have shown that the redox state of the pyridine nucleotide and flavoprotein pools reflects the balance between the rate of reduction by substrate utilization and the rate of oxidation by mitochondrial respiration. The shift of the redox balance towards a net reduced state occurs as a consequence of up-regulated substrate processing and inhibition of respiration, whereas an increase in mitochondrial respiratory rate favours the shift of the redox potential towards the oxidised state. Therefore it is conceivable that oxidised state of the pyridine nucleotide and flavoprotein pools in oocytes and zygotes from obese females may be attributable to increased mitochondrial respiratory chain activity.

**Figure 3:**
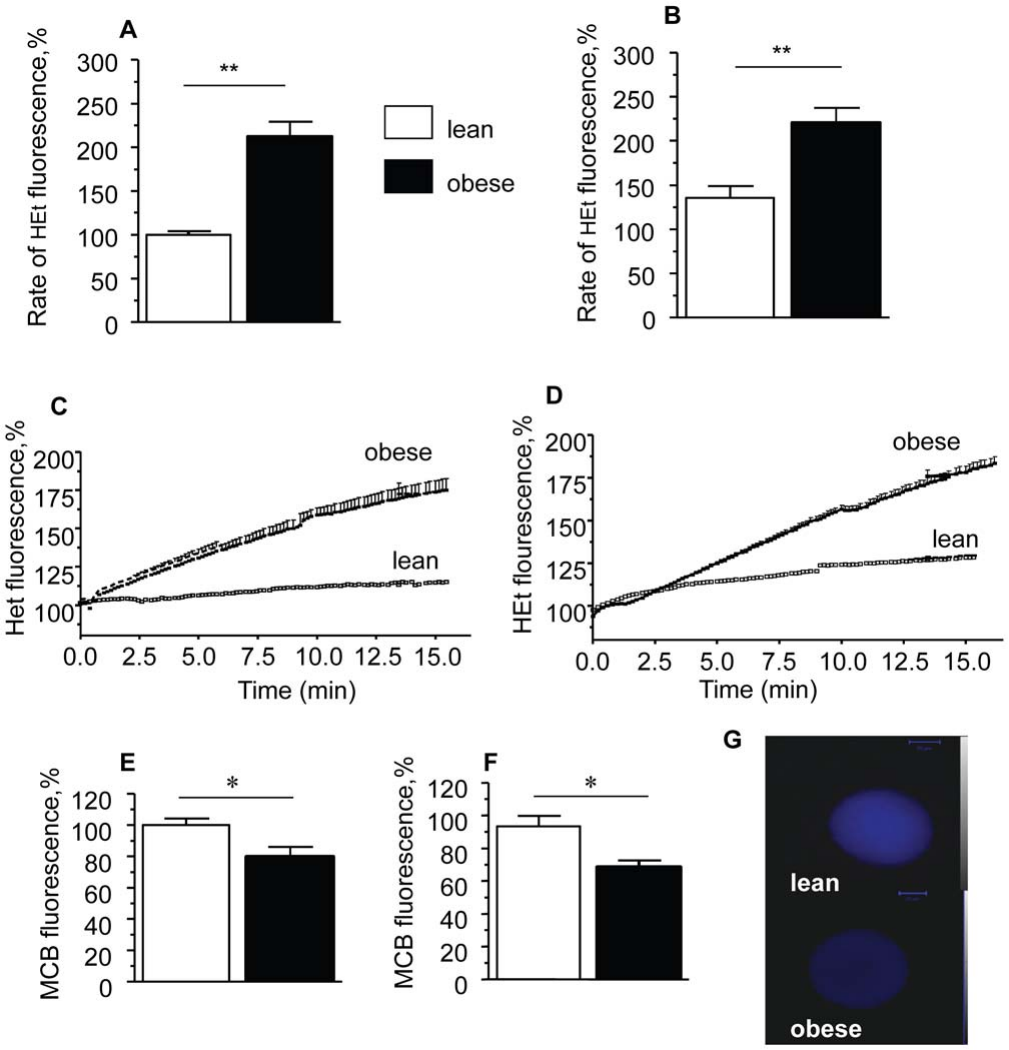
Maternal diet induced obesity increases rates of ROS generation and depletes glutathione in oocytes and zygotes. Cytosolic ROS production in oocytes and zygotes was measured by rate of oxidation of HEt. The traces represent changes of HEt fluorescence in oocytes (A) and zygotes (B) from lean (n = 15 cells/group) and obese (n = 15 cells/group) mice as a function of time. These data are summarised in histograms (C, D), in which the mean rates of ROS production are shown as the mean rate of HEt fluorescence change per minute. Results are expressed as percentage changes from HEt fluorescence in lean oocytes. Intracellular glutathione staining with MCB in oocytes (E) and zygotes (F) recovered from lean and obese female mice. Relative intensity of MCB fluorescence is expressed as a percentage of the signal from oocytes of lean mice. (G) Representative confocal images of GSH staining in oocytes. *p<0.05, ** *p*<0.01. Data are mean ± SEM.

In order to evaluate the level of oxidative stress and antioxidant defence in oocytes and zygotes we measured rates of intracellular ROS generation using dihydroethidium (HEt), a non-fluorescent derivative of ethidium which is oxidised to a fluorescent product by superoxide. In oocytes and zygotes of obese mice the rate of ROS production was significantly increased by 2.1 (p<0.05) and 1.6 (p<0.05) fold respectively compared to the eggs of lean mice (Figures 3A and 3B). We measured levels of the antioxidant glutathione (GSH) using the monochlorobimane (MCB) which forms a fluorescent adduct following an enzyme catalysed reaction with GSH. In parallel with the increase in the oxidative load, GSH was depleted in oocytes and zygotes from obese females when compared with eggs from lean females (oocytes – 80%±7 vs 100%±6, p<0.05; zygotes- 68%±4 vs 93%±6, p< 0.05) (Figures 3C and 3D).

Alterations in the rates of intracellular ROS generation are associated with changes in mitochondrial abundance and mtDNA copy number. Oxidative stress damages bases as well as causing single or double-strand breaks in mtDNA which are mutagenic and can inhibit mtDNA replication [27]. However, excessive ROS generation has been associated with an increase in mtDNA copy number in aging tissues as a result of a feedback response which compensates for defective mitochondria bearing impaired respiratory chain or mutated mtDNA [28].

**Figure 4:**
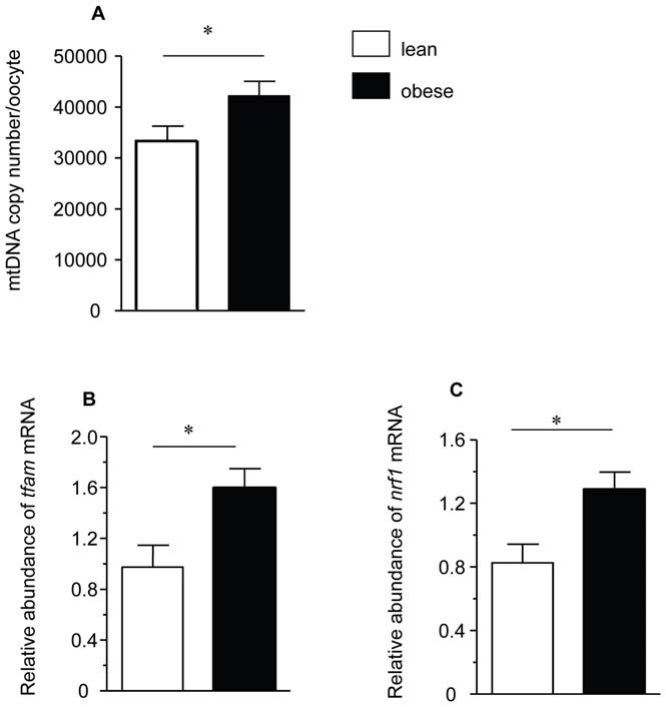
Mitochondrial biogenesis is up-regulated in oocytes from obese mice. (A) mtDNA copy number in oocytes from lean (n = 2 oocytes/8 females/group) and obese (n = 2 oocytes/8 females/group) mice. Relative abundance of *TFAM* mRNA (B) and *NR1m*RNA (C) in oocytes from lean (n = 20 oocytes/8 females/group) and obese (n = 20 oocytes/8 females/group) mice. qPCR was used to determine the absolute mtDNA copy number and the amount of specific transcripts relative to the *H2A*mRNA.* *p*<0.05. Data are mean ± SEM.

To test the hypothesis that maternal obesity-associated oxidative stress in oocytes and zygotes may affect mitochondrial biogenesis we measured mtDNA copy number and expression of key genes involved in the regulation of the replication and transcription of the mitochondrial genome. mtDNA copy number was significantly increased in oocytes from obese compared to lean mice (Figure 4A), and the expression of nuclear genes encoding mtDNA transcription factors - *mtTFAM* and *NRF1* - was also elevated in oocytes from obese females suggesting upregulation of mitochondrial biogenesis (Figure 4B and 4C). Interestingly, mtDNA copy number, *TFAM* and *NRF* 1 expression were not altered in zygotes from obese mice.

### Effects of maternal obesity on pre-implantation embryos

No significant differences were detected in the number of zygotes between lean and obese females. However, the ability of zygotes to develop to the blastocyst stage was reduced in obese mice (Table 2). Thus, the number of obese females who failed to produce blastocysts 4 days after mating was higher in comparison with controls (5 vs 2). In three of 5 obese mice no blastocyst were found in either uterus and oviduct whereas in two obese females a small number of arrested fragmented embryos at various stages of development was recovered from the oviduct. In the remaining 6 obese (54%) females blastocyst development per mouse was similar to that in lean females. Differential nuclear labeling did not reveal any differences in the number of cells within trophectoderm and inner cell mass lineages of blastocysts from obese and lean mice (Table 2).

**Table 2:** The influence of maternal diet –induced obesity on early embryo development.

Parameter	Control females	Obese females	Significance
Number of females with zygotes	100% (7)	100% (7)	P>0.05
Zygote recovered/mouse	7.9±0.1 (7)	8.9±1.7 (7)	P>0.05
Number of females with blastocysts/mated	82% (9/11)	54% (6/11)	
Number of females without blastocycts/mated 1	18% (2/11)	46% (5/11)	P<0.02 (11)
Blastocyst recovered/mouse	6.7±1.2 (7)	7.7±1.6 (6)	P>0.05
Blastocyst total cell number	45.8±2.1 (7)	44.8±3.2 (6)	P>0.05
ICM cell number	14.3±1.2(7)	14.1±0.6 (6)	P>0.05
TE cell number	31.7±1.6 (7)	30.6±0.8 (6)	P>0.05
ICM/TE cell number (%)	24.1±5.2 (7)	22.2±1.3 (6)	P>0.05

Data expressed as mean ± SEM. The number of zygotes was determined in the morning after natural mating. Blastocysts were recovered from the uterus on day 4 after mating. The number of cells per blastocyst and their distribution between the inner cell mass and the trophectoderm were analysed in 3–5 blastocysts per mouse. Values in parentheses indicate a number of mice/group.

1 In both control and in 2 out of 5 obese mothers a small number of fragmented embryos at various stages of development was recovered from the oviducts.

## Discussion

The negative impact of obesity on reproductive success is well documented [5] but the stages at which development of the conceptus is compromised and the mechanisms responsible for the developmental failure remain unclear. Using our established model we have identified altered mitochondrial activity as one of the probable mechanisms of obesity-associated reproductive and developmental failure.

We report that blastocyst development was reduced in maternal diet-induced obesity and was associated with altered mitochondrial distribution and striking hyperpolarisation of the mitochondrial membrane, oxidised redox state and oxidative stress in both oocytes and zygotes. Regulation of all of these parameters is required for normal development [13]. We also report increased mitochondrial biogenesis in oocytes as evidenced by high mtDNA copy number and up-regulation of *NRF1* and *TFAM* transcripts.

It is currently unclear what mechanisms underlie such large differences in Δψ between control oocytes and embryos and those derived from obese females. Nor it is entirely clear how these differences are established and maintained. Studies in mitochondria in somatic cells [29] and embryos [30], [31] have shown that the magnitude of Δψm is related to the level of mitochondrial respiration so the differences in mitochondrial respiratory activity may account for the hyperpolarisation observed. The increased oxidation of reducing equivalents - NAD(P)H and FADH_2_ in oocytes and zygotes from obese females have also provided indirect evidence for activation of mitochondrial respiratory activity. Carbohydrates and fatty acids are the principal substrates for mitochondrial oxidation and an increased availability of these energy substrates is a well known mechanism of up-regulation of mitochondrial respiration [32].The presence of excessive fuel within the obese reproductive environment may therefore increase substrate influx through the metabolic mitochondrial pathway, leading to activation of mitochondrial respiratory activity which is reflected in a hyperpolarised state of the mitochondrial membrane. Whether oocytes and embryos that show intense mitochondrial hyperpolarisation also have abnormally higher ATP content remain to be determined.

While the increase in Δψm in mouse oocytes and zygotes of obese mice may be due to increased energy substrate load to mitochondria [33], others have suggested that mitochondrial hyperpolarisation might be associated with early molecular events that precede developmental impairment and the induction of cell death [31]. Recently, Schienke et al. [34] has also reported reduced differentiation potential in mouse embryonic stem cells with a high Δψm and overall rate of mitochondrial metabolism.

Maternal diet-induced obesity was associated with increased oxidation of NAD(P)H in both oocytes and zygotes. NAD(P)H has direct antioxidant properties and also ensures regeneration of GSH, a developmentally critical antioxidant molecule [35]. Therefore, NAD(P)H down-regulation and reduced antioxidant capacity may contribute to oxidative stress in the oocytes and zygotes from obese females, as observed through direct measurements of ROS production and GSH concentration in the eggs. Unlike somatic cells, preimplantation embryos cannot synthesize GSH de novo [36] and may therefore be very sensitive to ROS, even at low concentrations [7].

ROS can be toxic when in excess but may also play a regulatory role in the control of mitochondria activity, particularly in mitochondrial biogenesis [37]. In cell lines carrying different common mouse mtDNA haplotypes a direct correlation between ROS generation and mtDNA content has been shown [38]. This is in agreement with our observation that oocytes from obese females producing more ROS had a higher mtDNA copy number than oocytes from lean mice. Expression of the nuclear genes involved in mitochondrial biogenesis, specifically *PGC-1*, *NRF-1* and *mtTFA* are up-regulated in some human cell types [39] and in rat hepatocytes [40] in response to oxidative stress. Hence, the increased mtDNA copy number as well as *NRF1* and *TFAM* mRNA expression in the oocyte of the obese female may be attributable to an oxidative stress-mediated increase in the transcription of genes involved in mitochondrial biogenesis. Surprisingly, increased mitochondrial biogenesis was present in the oocytes but not the zygotes of the obese females. Mitochondria replication and the synthesis of maternal nuclear encoded transcripts associated with mtDNA replication are ongoing processes in the growing oocyte [10] and therefore can be susceptible to ROS or nutritional and hormonal factors present in obese reproductive environment [12]. The normalisation of mtDNA and mRNAs associated with mtDNA replication after fertilization may be explained by a combination of our recent findings [15] and established molecular characteristics of the early embryo [41]. We have previously reported a period of mtDNA turnover where both mtDNA synthesis and destruction occur for a short period after fertilization [15]. This period of mtDNA turnover may offer a mechanism by which abnormal levels of mtDNA could be normalised in zygote stage embryos from obese dams. Additionally, it is well established that all maternally inherited transcripts are degraded during the 1–2 cell stage prior to the onset of the main burst of zygotic transcription during the late two cell stage [41]. Thus the normalisation of nuclear encoded transcripts associated with mitochondrial biogenesis in zygotes from obese reflects this documented global maternal mRNA destruction.

In this study a significant impairment in the ability to support embryo development to the blastocyst stage has been seen in 46% of the obese mothers. Similar numbers of zygotes were readily recovered from both groups, however. Thus, the absence of blastocysts in 46% of obese females is not attributable to anovulation but more likely due to increased embryonic death since fragmented embryos were found in the oviducts of 2 out of 5 obese females that failed to produce blastocysts. In support of this idea, a recent study by Minge et al [42] has reported poor oocyte quality, reduced blastocyst survival rates and abnormal embryonic cellular differentiation in obese female mice. In women obesity is also associated with poor pre-implantation embryo quality and a reduced rate of embryo survival [5].

We also found that the obesogenic diet increased serum fatty acid concentration and caused a marked increase in the oviductal leptin concentration. Leptin is essential for normal pre-implantation development of mouse embryos [43]. However, exposure to a higher leptin concentration *in vitro* [44] and *in vivo* [45] impairs embryo development, reducing the rate of blastocyst formation. In obese women, hyperleptinaemia is associated with poor fertility and increased risk of early pregnancy loss [46]. Leptin is also crucial to mitochondrial function. In rodents, leptin increases the expression of enzymes contributing to fatty acid oxidation [47] and up-regulates mitochondrial biogenesis through expression of PPARγ coactivator-1α [48]. Hyperlipidemia, also frequently associated with obesity, increases fatty acids flux into oocytes and embryos which may increase ROS generation or trigger embryo apoptosis through mitochondria–dependent pathways [49].

In conclusion, exposure of oocytes and embryos to an obese reproductive environment was associated with qualitative and quantitative changes in mitochondria, oxidised redox state, increased oxidative load and impaired antioxidant capacities. Oocytes and embryo with compromised mitochondrial activity may not be able to exert tight regulation of focal substrate supply and demand and, as a result, generate ROS at rates that become developmentally toxic after fertilization [7]. Embryo apoptosis or arrest may then ensue. Further investigation of mitochondrial functions in oocytes and embryos are required, particularly with regard to obesity-related alterations in mitochondrial gene and protein expression that inappropriately regulate mitochondrial energy metabolism.

We propose that altered oocyte and early embryo mitochondrial metabolism, resulting from excessive nutrients exposure prior to and during conception may be responsible for poor reproductive outcomes frequently reported in obese women.

## Materials and Methods

### Experimental animals and diets

This study was conducted in accordance with the UK Home Office Animal (Scientific Procedures) Act 1986. Six week-old virgin females C57BL/6J mice (Charles River Laboratories, UK) were fed either a standard chow diet (7% simple sugars, 3% fat, 50% polysaccharide, 15% protein [w/w] RM1, Special Dietary Services, n = 10) or highly palatable obesogenic diet (10% simple sugars, 20% animal lard, 28% polysaccharide, 23% protein [w/w], Special Dietary Services, n = 10) supplemented with sweetened condensed milk and micronutrient mineral mix (AIN93G, Special Dietary Services) [19]. After 6 weeks of diet, all females were induced to superovulate by consecutive ip injections of 10 IU pregnant mare's serum (Dunlops) and 10 IU human chorionic gonadotrophin (hCG, Dunlops). Prior to embryo collection mice were culled by cervical dislocation. Blood samples were taken via cardiac puncture. Abdominal and inguinal fat pads and body weights were recorded. Twelve hours post hCG fully grown oocytes were collected by puncturing pre-ovulatory follicles with sterile needles and treated with hyaluronidase (0.5 mg/ml) to remove surrounding cumulus cells. Zygotes and blastocysts were collected after successful mating with males at 24 and 84 h after post-hCG as described [15].

Oviduct fluid was collected as described in [50] with minor modifications. Briefly, pregnant female mice (day 4) were culled and oviducts were ligatured at uterotubal junction with 6-0 black suture silk. Oviducts with attached ovaries were excised, rinsed in saline and placed under mineral oil on a watch glass. Ovaries were separated and the thread was removed at the cut end of the uterus under the microscope. A curved blunt-end metal capillary connected to a 1-ml syringe was inserted into infundibulum and oviducts were carefully flushed with 50 µl of flushing medium (PBS supplemented with 0.3% polyvinylpyrrolidone). Flushing from both oviducts was collected and placed into a sterilized 0.5-ml microcentrifuge tube and centrifuged at 10,000×g for 10 min to remove cellular debris, and the supernatant was aspirated into another sterilized 0.5-ml microcentrifuge tube and stored at −80°C until assayed for concentrations of leptin and glucose.

### Assessment of mitochondrial morphology and metabolism

Mitochondria of living oocytes and zygotes were imaged using a Zeiss 510 uv-Vis CLSM META and a range of targeted fluorescent probes (Molecular Probes) as described previously [23], [51]. Some measurements were made using a cooled CCD camera (Orca ER).

The distribution of active mitochondria and Δψ_m_ were analysed in eggs incubated with tetramtehyl rhodamine methyl ester (TMRM; 25 nM) in M2 medium at 37°C for 30 min. The TMRM is a fluorescent lipophilic cation and accumulates into mitochondria in response to the negative mitochondrial membrane potential. TMRM was excited using the 543 nm laser line and fluorescence measured using a 560 long-pass filter.

Measurements of NAD(P)H and FAD^2+^ autofluorescence intensity in oocytes and zygotes were used to estimate the mitochondrial redox potential. The reduced forms of pyridine nucleotides (NAD(P)H) are excited by ultraviolet light at 351 nm excitation line of the CLSM and measured between 435–485 nm. The oxidised form (NAD(P)^+^) is non-fluorescent. In contrast to NAD(P)H, it is the reduced form of flavoproteins (FADH_2_) that is non-fluorescent. Fluorescence of the oxidised form of flavoproteins (FAD^2+^) was excited at 458 nm and emitted fluorescence was collected throughout the 505–550 nm bandpass filter.

The resting redox state was defined as a function of the maximally oxidised and maximally reduced signals which were obtained by adding FCCP (1 µM) to drive the signals to maximal oxidation followed by addition of 1 mM NaCN which drives the signals to a maximally reduced state. The fluorescence signals are then normalised between 100 (maximal reduction to NAD(P)H and maximal oxidation to FAD^2+^) and 0 (maximal oxidation to NAD(P)^+^ and maximal reduction to FADH_2_), giving a value which is a measure of the resting relative redox state [23].

For measurement of cytosolic ROS production, HEt (2 µM) was added to M2 medium and remained present throughout the experiment (15 min). This is a non-fluorescent derivative of the red fluorescent ethidium, and so an increase in red fluorescence (excited at 543 nm and measured at >560 nm) gives a measure of the rate of oxidation of the dye and therefore of the rate of ROS generation.

In order to measure GSH, cells were incubated with 50 µM MCB in M2 medium at 37° for 40 min, or until a steady state had been reached before images were acquired. Non-fluorescent MCB undergoes a reaction with glutathione catalysed by glutathione-s-transferase to yield a fluorescent adduct which therefore gives a measure of GSH content [52]. MCB fluorescence was excited at 351 nm and measured at 430–480 nm.

Fluorescent images were obtained from at least from 3 oocytes/zygotes from 5 females per diet. Image analysis, differentiation and exponential curve fitting were performed using Origin 8 software (OriginLab Corporation).

### Quantification of mDNA copy number

Total DNA was extracted from groups of two oocytes and zygotes (n = 8 females/group) as described [53]. A mtDNA content was measured by qPCR on Thermal Cycler Corbett Rotorgene ™ 6000 (Corbett research) using QuantiFast SYBR Green PCR Kit (Qiagen) and primers corresponding to *16S* ribosomal gene. Sample copy number was determined with mtDNA standard curves using Rotorgene 6000 software.

### Gene expression analysis

Poly(A)+ RNA was isolated from snap-frozen oocytes (n = 20 oocytes/8 females/group) and zygotes (n = 10zygotes/8 females/group) using magnetic oligo(dT) beads (Dynabeads mRNA DIRECT Kit). cDNA synthesis was performed by random hexamer priming and the Transcriptor First Strand cDNA Synthesis Kit (Roche). qPCR took place on a Chromo4 thermocycler (MJ) using the Precision SybrGreen Master Mix (Primerdesign). Each assay was performed in duplicates using intron-spanning primers (Operon Biotechnologies GmbH). Stability of housekeeping genes was validated using geNorm application and *H2afz* mRNA was selected for normalization. Relative mRNA abundance was determined using the comparative deltaCt method. A list of genes investigated and Primer sequences are shown in Table 3.

**Table 3:** Genes investigated and relevant primer sequences.

Gene	Forward primer	Reverse primer	Reference sequence
TFAM	CAA AGG ATG ATT CGG CTC AG	AAGCTGAATATATGC CTGCTTTTC	NM_009360
NRF1	GTC ACC ATG GCC CTC AAC	GGA CTA TCT GTC TCC CAC CTTG	NM_010938
H2afz	ACA GCG CAG CCA TCC TGG AGT A	TTC CCG ATC AGC GAT TTG TGG	NM_016750
16SrRNA	CTGCCCAGTGACTAAAGTTTAACG	CGTTCATGCTAGTCCCTAATTAAGGA	V00665

### Differential nuclear staining

The number of cells per blastocyst and their distribution between the inner cell mass and the trophectoderm were counted by differential fluorochrome nuclear labelling [54].

### Metabolic Studies

Fasted glucose, fatty acids and triglycerides concentrations were assessed using autoanalyser (LX20, Beckman Coulter) and the assay kits (Glucose; UV-hexokinase; nr. GLU 1442640; triglycerides; enzymatic GPO method; nr. TG 445850; total cholesterol; enzymatic method; nr. CHOL 467825) as described in [19]. Concentrations of leptin were measured by a sandwich ELISA using paired leptin antibodies (Duoset, R&D Systems Ltd). Serum and oviduct fluid samples from the mice were diluted 1∶20 for the assay and leptin concentration of samples was calculated from a standard curve constructed with mouse leptin standards.

### Statistics

All results are expressed as mean ± SEM. Data were analysed by Student's t-test after testing for normal distribution using Graphpad Prism v. 2.01 (Graphpad Software, USA). A value of p<0.05 was considered significant.
